# Plasticity of Dopaminergic Phenotype and Locomotion in Larval Zebrafish Induced by Brain Excitability Changes during the Embryonic Period

**DOI:** 10.1523/ENEURO.0320-21.2023

**Published:** 2023-06-26

**Authors:** Sandrine Bataille, Hadrien Jalaber, Ingrid Colin, Damien Remy, Pierre Affaticati, Cynthia Froc, Jean-Pierre Levraud, Philippe Vernier, Michaël Demarque

**Affiliations:** 1 Université Paris-Saclay, Centre National de la Recherche Scientifique, Institut des Neurosciences Paris-Saclay, Saclay 91400, France; 2 Université Paris-Saclay, Centre National de la Recherche Scientifique, Institut national de recherche pour l'agriculture, l'alimentation et l'environnement, Trangénèse pour les Etudes Fonctionnelles chez les ORganismes modèles Paris-Saclay, Saclay 91400, France; 3 Université Paris‐Saclay, Centre National de la Recherche Scientifique, Institut Pasteur, Université Paris‐Cité, Institut des Neurosciences Paris‐Saclay, Saclay 91400, France

**Keywords:** differentiation, dopamine, locomotion, plasticity, specification, zebrafish

## Abstract

During the embryonic period, neuronal communication starts before the establishment of the synapses with alternative forms of neuronal excitability, called here embryonic neural excitability (ENE). ENE has been shown to modulate the unfolding of development transcriptional programs, but the global consequences for developing organisms are not all understood. Here, we monitored calcium (Ca^2+^) transients in the telencephalon of zebrafish embryos as a proxy for ENE to assess the efficacy of transient pharmacological treatments to either increase or decrease ENE. Increasing or decreasing ENE at the end of the embryonic period promoted an increase or a decrease in the numbers of dopamine (DA) neurons, respectively. This plasticity of dopaminergic specification occurs in the subpallium (SP) of zebrafish larvae at 6 d postfertilization (dpf), within a relatively stable population of vMAT2-positive cells. Nondopaminergic vMAT2-positive cells hence constitute an unanticipated biological marker for a reserve pool of DA neurons that can be recruited by ENE. Modulating ENE also affected larval locomotion several days after the end of the treatments. In particular, the increase of ENE from 2 to 3 dpf promoted hyperlocomotion of larvae at 6 dpf, reminiscent of zebrafish endophenotypes reported for attention deficit hyperactivity disorders (ADHDs). These results provide a convenient framework for identifying environmental factors that could disturb ENE as well as to study the molecular mechanisms linking ENE to neurotransmitter specification.

## Visual Abstract

**Figure:**
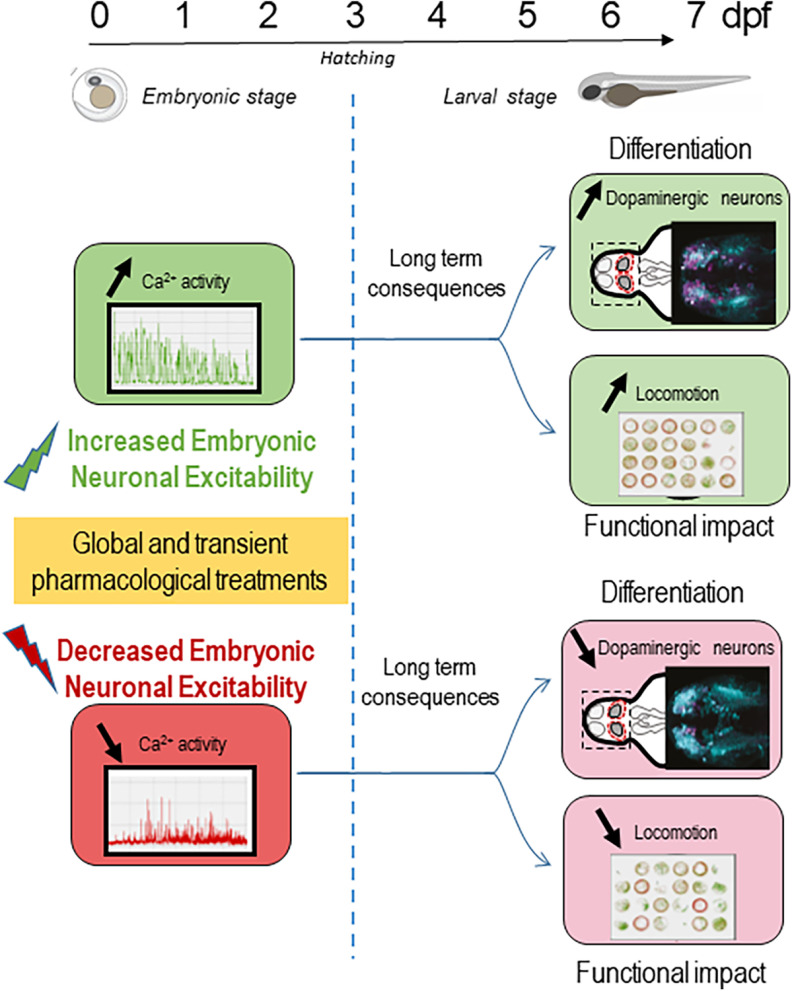

## Significance Statement

Spontaneous calcium (Ca^2+^) transients, used here as a proxy for embryonic neural excitability (ENE), are detected in the forebrain of embryonic zebrafish. Using short-term pharmacological treatments by bath application that increase or decrease ENE we detected changes in the postmitotic differentiation of the dopaminergic phenotype, occuring within a reserve pool of vMAT2-positive cells. We also report changes in locomotion, with transient increase of ENE leading to hyperlocomotion, a phenotype associated with attention deficit hyperactivity disorder (ADHD) in this model. Our results provide a convenient framework to study the molecular mechanisms linking ENE to neurotransmitter specification.

## Introduction

During brain development, specific molecular components of synaptic neuronal communication become functional before synapse formation, such as voltage-sensitive ion channels and neurotransmitter receptors at the plasma membrane, and the release of neurotransmitters in the extracellular space (Spitzer et al., 2002). They contribute to immature forms of cellular excitability and intercellular communications that we refer to here as embryonic neural excitability (ENE). There is for instance a paracrine communication mediated by nonsynaptic receptors activated by endogenous neurotransmitters, in the neonatal rat hippocampus, and in the mouse spinal cord (Demarque et al., 2002; Owens and Kriegstein, 2002; Scain et al., 2010). Acute changes of calcium (Ca^2+^) concentration with different spatiotemporal dynamics have also been described in differentiating neurons, either locally, in filopodia or growth cone or globally, at the level of the soma (Gomez and Spitzer, 1999; Gomez et al., 2001). The latter, that we refer to here as Ca^2+^ transients, are sporadic, long-lasting global increases of intracellular concentration of Ca^2+^ that occur during restricted developmental windows called “critical periods.” They have been identified in the developing brain of several vertebrate species (Owens and Kriegstein, 1998; Crépel et al., 2007; Blankenship and Feller, 2010; Demarque and Spitzer, 2010; Warp et al., 2012). Changes in the incidence and frequency of Ca^2+^ transients have been shown to modulate the specification of the neurotransmitter phenotype in various populations of neurons, as is the case for dopamine (DA), in the *Xenopus* and rat brain, with consequences on several behaviors (Dulcis and Spitzer, 2008; Dulcis et al., 2013, 2017).

DA is an evolutionarily conserved monoamine involved in the neuromodulation of numerous brain functions in vertebrates, including motivational processes, executive functions, and motor control (Klein et al., 2019). Accordingly, alterations of the differentiation and function of the neurons synthesizing DA contribute to the pathogenesis of several brain diseases with a neurodevelopmental origin, such as attention deficit hyperactivity disorder (ADHD) and schizophrenia (SZ; Lange et al., 2012; Murray et al., 2017).

Further addressing the complex molecular and cellular mechanisms linking developmental excitability, dopaminergic differentiation, and behavioral outputs requires an *in vivo* approach in an accessible and genetically amenable animal model. The developing zebrafish *Danio rerio* fits these needs. The development of the embryo is external, which allows perturbing ENE at stages that would be *in utero* in mammalian models. The embryos are relatively small and transparent, simplifying high-resolution imaging of the brain in live or fixed preparations. Despite differences in brain organization, notably a pallium very different from the six-layered cortex of mammals, the main neuronal systems of vertebrates are present in zebrafish and respond to psychoactive drugs (Gawel et al., 2019). In addition, the monoaminergic system has been extensively studied in zebrafish (Schweitzer and Driever, 2009; Schweitzer et al., 2012). In vertebrates, DA modulates executive functions, such as working memory and decision-making, through the innervation of specific regions of the telencephalon. In the zebrafish brain, most DA neurons innervating the telencephalon have their cell bodies located within the telencephalon itself (SP-DA cells; Tay et al., 2011; Yamamoto et al., 2011). To the best of our knowledge, the plasticity of the neurotransmitter phenotype in these cells has not been studied so far.

To perturb ENE globally, we used pharmacological treatments by bath application from 48 to 72 h postfertilization (hpf). We then analyzed the consequences of these transient pharmacological treatments a few days later, between 6 and 7 d postfertilization (dpf). We report quantifiable changes in the specification of the dopaminergic phenotype in SP-DA neurons. We also report induced changes in locomotion within the same time frame. These results suggest a contribution of ENE to the specification of the dopaminergic phenotype and the establishment of motor control in the zebrafish. They also open important perspectives for this model to decipher the molecular events leading from environmental factors to the modifications of developmental trajectories.

## Materials and Methods

### Fish strains

All experiments were conducted following animal care guidelines provided by the French ethical committee and under the supervision of authorized investigators.

Zebrafish were raised according to standard procedures (Westerfield, 2000). Briefly, for breeding, male and female zebrafish were placed overnight, in different compartments of a tank with a grid at the bottom that allows the eggs to fall through. The next morning the separation was removed and after a few minutes, the eggs were collected, rinsed, and placed in a Petri dish containing embryo medium (EM). Embryos were kept at 28°C, then staged according to standard criteria. The number of animals used for each experiment is indicated in the corresponding figure legends.

Wild-type zebrafish were of AB background. The following transgenic zebrafish lines were used: *Tg(hsp70l:Gal4)^kca4^, Tg(UAS:GCaMP6f;cryaa:mCherry)^icm06^, Tg(tbp:Gal4;myl7:cerulean)^f13^, Tg(elalv3:Gal4)^zf34^*, and *Tg(Et.slc18a2:GFP)^zf710^*.

### Pharmacological treatments

Pharmacological compounds, veratridine (10 μm), tetrodotoxin (TTX; 2 μm), ω conotoxin (0.08 μm), nifedipine (0.4 μm), and flunarizine (2 μm) were purchased from R&D Systems and prepared in water except for veratridine which required dimethyl sulfoxide (DMSO) for dissolution and flunarizine that requires ethanol (EtOH) for dissolution. Control exposures were performed using the same concentration of DMSO in EM without the drug. The specific period and duration of applications are indicated in the corresponding figure legends. All pharmacological treatments were performed by bath application followed by three washes in EM. Embryos were randomly distributed in wells (30 embryos per well) of a six-well plate containing 5 ml of solution (EM+DMSO or EM+drug). Embryos exposed to drugs or the control solution were observed for morphologic abnormalities every day until 5 dpf. Malformations (e.g., spinal curvature, cardiac edema) were considered experimental end-points, and when detected the corresponding animals were excluded from the study.

### Calcium imaging

For Ca^2+^ imaging, we measured the fluorescence of the genetically encoded Ca^2+^ sensor GCaMP6f, expressed under the control of a UAS promoter (*UAS:GCaMP6f*). We used three different gal4 lines to drive UAS-dependent expression of GCaMP: a (*TBP:Gal4*) line, in which the TATA-box binding protein (TBP) promoter drives constitutive ubiquitous expression of the transgene, a Tg(hsp70l:Gal4) line in which the expression is trigerred following a transient increase in temperature, and a (*Elavl3:Gal4*) line, in which the Elavl3 promoter drives pan-neuronal expression of the transgene.

At 24 hpf, embryos of the (*Hsp70:GAl4;UAS:GCaMP6f*) line were exposed to a 38°C temperature for 1.5 h. Upon heat shock activation, GCaMP6f is expressed in all the cells of the animals and remains detectable for several days.

At 2–3 dpf, the embryos were paralyzed with intramuscular injections of 750 μg/ml α-bungarotoxin (Life Technologies), then individually embedded in low melting agarose (Life Technologies), ventral side up, for imaging.

Thirty-minute time-lapse series were acquired at 1 Hz, at a single focal plane, on an Olympus BX60 microscope (Olympus Corporation) equipped with a 40 × 0.6 N water immersion objective. A nonlaser spinning disk system (DSD2, ANDOR Technology) was used for illumination and image acquisition. Images were processed with Fiji. Movements of the preparation in the *x*-/*y*-axis were corrected using the “Stackreg” plugin (W. Rasband, B. Dougherty). Regions of interest (ROIs) were drawn manually over individual cell bodies and the average gray level from pixels in ROIs was measured over time using the MultiMeasure plugin (Optinav). Sequential values of fluorescence were then treated in MATLAB (MathWorks). Transients were defined as an increase of fluorescence higher than 2.5 times the standard deviation of the baseline. The duration and amplitude of transients were calculated using the “peak” function. Incidence was scored as the number of cells generating transients divided by the estimated total number of cells in the imaged field and was expressed as a percentage. Frequency was calculated as the total number of transients in a given cell divided by the total acquisition time and was expressed as transients per hour.

### Immunohistochemistry

#### Tissue preparations

Six- to 7-dpf zebrafish larvae were deeply anesthetized using 0.2% Ethyl3-aminobenzoate methanesulfonate (MS222; Merck KGaA) diluted in EM, then they were fixed in ice-cold 4% paraformaldehyde (PFA; Electron Microscopy Sciences) in 1× PBS (Fisher Scientific) containing 0.1% Tween 20% (PBST) overnight at 4°C. Samples were dehydrated and stored in MeOH at −20°C.

#### Immunofluorescence

Immunofluorescence was performed in 2-ml microtubes. Unless specified otherwise in the protocol, incubations were performed at room temperature (RT), and thorough PBST washes were performed between each step. The samples were first incubated in 3% hydrogen peroxide (H_2_O_2_) solution in ethanol (EtOH) 100% for 30 min, to deactivate endogenous peroxidases. They were then successively incubated in EtOH:Xylene 1:1 without agitation for 1 h and, at −20°C, in EtOH:acetone 1:2 without agitation for 20 min. The washes performed between these steps were performed in EtOH. After the final wash, samples were rehydrated in PBST.

To unmask the antigens, samples were incubated in PBST:Tris 150 mm pH 9 for 10 min, then in Tris 150 mm pH 9 at RT for 10 min and at 70°C for 30 min. After PBST washes, the samples were incubated in blocking buffer 1 [10% normal goat serum (NGS), 1% Triton X-100, 1% Tween 20, 1% DMSO, 1% bovine serum albumin (BSA) in PBS 1×] for 3 h.

Two protocols were used for primary antibody staining, adapted from published protocols (Inoue and Wittbrodt, 2011; Xavier et al., 2017; Bloch et al., 2019). For the TH antibody, the samples were incubated with the first primary antibody (mouse anti-TH, 1:250; Yamamoto et al., 2010) in primary staining solution (1% NGS, 1% Triton X-100, 1% DMSO, 1% BSA, 0,05% azide sodium in PBST) at 4°C for 7–10 d, under gentle agitation. After washing, the samples underwent a step of refixation in PFA 4% for 2 h at RT and were washed overnight in PBST.

The samples were then incubated in blocking buffer 2 (4% NGS, 0,3% Triton X-100, 0.5% DMSO in PBST) for 1 h at RT and incubated with a first secondary antibody (anti-mouse biotinylated, 1:200; Alunni et al., 2013) in secondary staining buffer (4% NGS, 0,1% Triton X-100 in PBST) for 2.5 d at 4°C under gentle agitation.

For the revelation, we used the Vectastain ABC kit (Vector). Briefly, the AB mix was prepared by adding 10 μl of solution A and 10 μl of solution B in 1 ml PBST/1% Triton X-100. One hour after the preparation, the samples were incubated in the AB mix for 1 h. Samples were then incubated in Tyramide-TAMRA (1:200 in PBST) for 20 min, then 0.012% H_2_O_2_ was added directly to the solution and the samples were incubated for an additional 50 min.

Before a second primary antibody incubation, the samples underwent a step of fixation in PFA 4% for 2 h at RT and were washed overnight in PBST.

For the other primary antibodies (rabbit anti-caspase3, 1:500 or chicken anti-GFP, 1:500; Xavier et al., 2017), the samples were incubated in blocking buffer 1 for 3 h, then were incubated with the primary antibody in primary staining solution at 4°C for 3–4 d, under gentle agitation. After washes and refixation as above, the samples were incubated with the second secondary antibody (goat anti-chicken Alexa Fluor 488, 2 μg/ml) and DAPI 1× in PBST at 4°C for 2.5 d under gentle agitation. Samples were then washed three times in PBST and left overnight in PBST. For observation, brains were dissected and mounted between slides and coverslips in Vectashield solution (Vector).

### Image acquisition

A Leica TCS SP8 laser scanning confocal microscope with a Leica HCTL Apo × 40/1.1 w objective was used to image the specimens.

The fluorescence signal was detected through laser excitation of fluorophores at 405, 488, 552, or 638 nm and detection was performed by two internal photomultipliers. Steps in the *z*-axis were fixed at 1 μm. Acquired images were adjusted for brightness and contrast using ImageJ/FIJI software.

### Quantification of immunoreactive cells

The R software “sample” function was used to attribute a random number to each sample allowing for counting by observers blinded to the treatment group. The TH-immunoreactive and GFP-immunoreactive cells were counted manually from z-stacks of confocal images using the ImageJ cell counter plugin.

### Spontaneous locomotion assays

We recorded the locomotion of individual larvae at 6–7 dpf, a period of relative stability for swimming parameters (Colwill and Creton, 2011; data not shown). larvae were placed in wells of 24-well plates with 2 ml of EM 2 h before the recordings for habituation. The plate were then placed on an infrared floor, under an infrared-sensitive camera (Zebrabox, Viewpoint) for 10-min recording sessions. Locomotor activity was recorded using ZebraLab software (Videotrack; ViewPoint Life Sciences). We first performed sessions with anesthetized larvae using several thresholds to help determine the minimum value allowing removing noise from the movement dataset. A 1.5 mm.s^−1^ low limit led to the elimination of >90% of quantification not related to active specimen movement (data not shown). This value was used as a low threshold in subsequent analysis.

One characteristic of larval swimming behavior is the presence of high-speed swimming episodes called bursts. They are classically defined as swimming episodes with a speed higher than 20 mm.s^−1^ (Budick and O’Malley, 2000). We therefore used this value as a high threshold to distinguish burst from cruise episodes in our analysis.

For the experimental sessions, we assessed the distance covered by the larvae without using thresholds. We then analyzed the distance covered during cruises, i.e., during episodes with a speed between 1.5 and 20 mm.s^−1^ and the distance covered associated with bursts, i.e., during episodes with a speed >20 mm.s^−1^. Each data point correspond to the distance covered by a larva during the entire experimental session.

### Statistical analyses

Results are shown as scatterplots overlaid with box and whisker plots showing minimum (bottom whisker), maximum (top whisker), mean (cross), median (line), first quartile (bottom of the box), and third quartile (top of the box), and each value (dots). For means comparisons, we first performed the Shapiro–Wilk test for normality. If all samples passed the normality test, we then checked for equality of variance. When all samples had similar variances, we performed an ordinary ANOVA test. When differences in variances were detected, we performed Brown–Forsythe and Welch ANOVA tests (BFW ANOVA). Multiple comparisons were then performed using Dunnett’s test. If one sample or more did not pass the normality test, we used the nonparametric Kruskal–Wallis test coupled with Dunn’s test for multiple comparisons (KW). Tests and *p* values are in the figure legends; *p* < 0.05 was considered as the level for significance and is reported as follows on graphs: *p < 0.05, **p < 0.01, ***p < 0.001, ****p < 0.0001 (ns = non significant).

Statistical tests were performed using Prism (GraphPad), except for the equality of variance test performed with the online software Brightstat (Stricker, 2008).

## Results

### Timing of experiments

**Figure 1.**
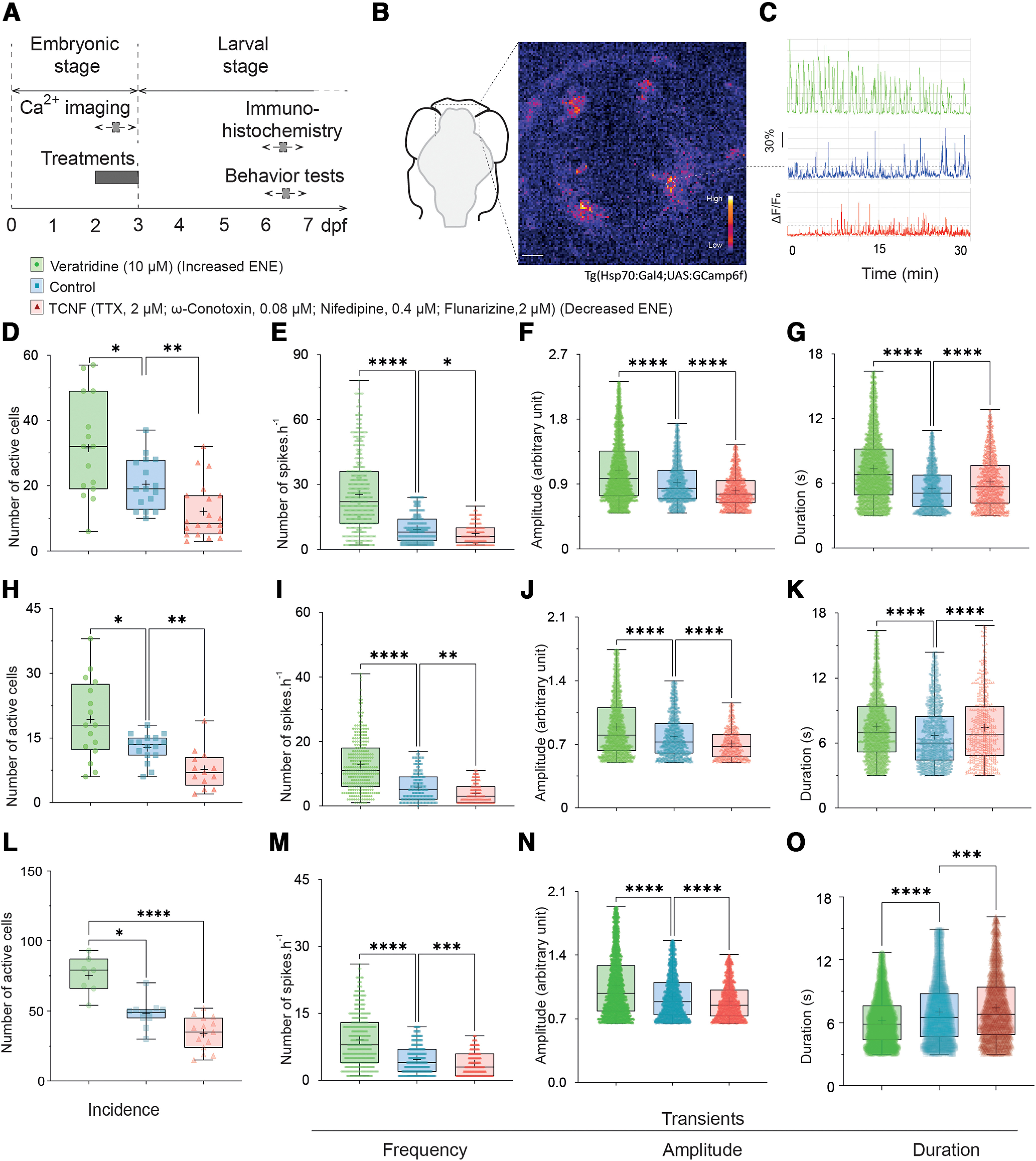
Timing of the experiments, characterization of forebrain calcium transients and their modification by bath application of pharmacological treatments. ***A***, Timeline of the experiments. Pharmacological treatments and calcium imaging experiments were performed from 2 to 3 dpf (embryonic period) and the immunohistochemistry and behavior experiments were performed several days later, at 6–7 dpf (larval period). ***B***, Left panel, Schematic ventral view of a zebrafish brain, showing the approximate region for calcium recordings. Right panel, Confocal image from a time-lapse recording of the brain of 48 hpf of *Et(hsp:gal4;UAS:GCamp6f)* embryos in control conditions. Fluorescence is displayed on a pseudocolor scale, the lookup table coding for the intensity scale is shown in the bottom-right corner of the image. Scale bar = 100 μm. White dash-circle defines an example region of interest corresponding to the cell body of a cell which changes in fluorescence are displayed in ***C***. ***C***, Representative changes in fluorescence intensity plotted as a function of time in different experimental conditions: control conditions in blue, following veratridine treatment (10 μm) in green, and following TCNF treatment (TTX, 2.5 μm; ω-conotoxin, 0.1 μm; nifedipine, 0.5 μm and flunarizine, 2.5 μm) in red. Ca^2+^ transients were scored as changes in fluorescence more than two times higher than the SD of the baseline (dashed lines), and >3 s in duration, calculated as the width at half-maximum. ***D–O***, Boxplots showing different parameters of calcium spikes in control conditions (blue), following 10 μm veratridine treatment (green) and following TCNF treatment (red). ***D–G***, Results obtained in the Hsp:gal4 line from five independent experiments. ***H–K***, Results obtained in the *TBP:gal4* line, from six independent experiments. ***L–O***, Results obtained in the *HuC:gal4* line, from three independent experiments. ***D***, Average incidence of the recorded Ca^2+^ transients, BFW ANOVA, 15 < *n* < 21 fields of view. ***E***, Average frequency, KW test, 291 < *n* < 535 values. ***F***, Average normalized amplitude of the recorded Ca^2+^ transients, KW test, 1258 < *n* < 4809 transients. ***G***, Average duration of the Ca^2+^ transients, KW test, 1258 < *n* < 4809 transients. ***H***, Average incidence of the recorded Ca^2+^ transients, BFW ANOVA, 13 < *n* < 17 fields of view. ***I***, Average frequency, KW test, 148 < *n* < 281 values. ***J***, Average normalized amplitude of the recorded Ca^2+^ transients, KW test, 607 < *n* < 2980 transients. ***K***, Average duration of the Ca^2+^ transients, KW test, 607 < *n* < 2980 transients. ***L***, Average incidence of the recorded Ca^2+^ transients, BFW ANOVA, 7 < *n* < 15 fields of view. ***M***, Average frequency, KW test, 507 < *n* < 527 values. ***N***, Average normalized amplitude of the recorded Ca^2+^ transients, KW test, 2442 < *n* < 5149 transients. ***O***, Average duration of the Ca^2+^ transients, KW test, 2442 < *n* < 5149 transients. *p < 0.05, **p < 0.01, ***p < 0.001, ****p < 0.001, ns = non significant.

To study the contribution of embryonic neural excitability (ENE) to zebrafish larval development, we adapted the transient pharmacological treatments previously validated in *Xenopus* (Borodinsky et al., 2004). Bath treatments were performed during the last day of embryonic development (48–72 hpf) to avoid effects on early developmental steps such as neurulation. The acute effect of the treatments were assessed by calcium imaging during the course of the treatments, while their long-term effects were studied by immunohistochemical and behavioral analysis at 6–7 dpf, several days after the washes (Fig. 1*A*). To increase ENE we used veratridine (10 μm), which blocks the inactivation of voltage-dependent sodium channels. To decrease ENE, we used a cocktail containing TTX (2 μm), ω-conotoxin (0.08 μm), nifedipine (0.4 μm), and flunarizine (2 μm) targeting voltage-dependent sodium channels, N, L, and T subtypes of voltage-dependent Ca^2+^ channels, respectively. We refer to this cocktail as TCNF.

### Spontaneous calcium transients in the brain of zebrafish embryos

To evaluate the level of ENE in the embryonic zebrafish brain, we used Ca^2+^ transients as a proxy. We followed the dynamics of intracellular Ca^2+^ concentration using time-lapse imaging of 2- to 3-dpf zebrafish embryos expressing the genetically encoded Ca^2+^ reporter GCaMP6f (Fig. 1*B*,*C*). Because of the noncell autonomous mechanism involved in the plasticity of the neurotransmitter phenotype described in *Xenopus* (Guemez-Gamboa et al., 2014), we used ubiquitous reporters to detect changes in fluorescence in all telencephalic cells. To induce UAS-dependent GCaMP6f expression, we first used the (*Hsp70:GAl4 x UAS:GCaMP6f;cry:mCherry*) line, in which the transgene is expressed on heat shock activation (see Materials and Methods; Fig. 1*D–G*). To ensure that the observed phenotypes were not altered by the initial heat shock, we confirmed these results using a *TBP:Gal4* driver, in which the TATA-box binding protein (TBP) promoter (Burket et al., 2008) drives constitutive transgene expression (Fig. 1*H–K*). To confirm that neurons were contributing to a majority of the pattern observed we repeated the experiments using a pan-neuronal reporter (elavl3:Gal4; Fig. 1*L–O*).

To monitor changes in fluorescence over time, we imaged of the anterior-most part of the brain of 2- to 3-dpf zebrafish embryos for 30-min sessions. We analyzed the evolution of the fluorescence from selected regions of interest (ROIs) corresponding to individual cell bodies. We used four parameters to analyze the results, the average frequency of transients per ROI, the incidence of active ROIs in the field of view (number of ROIs displaying at least one transient during the recordings over the estimated total number of ROIs), and the duration and amplitude of individual transients (Materials and Methods). In control conditions, we detected the presence of sporadic spontaneous transients in the telencephalon of the three transgenic backgrounds we used (Fig. 1*D–O*, blue symbols). The analyzed parameters were in the range of what has been reported in the *Xenopus* brain (Dulcis and Spitzer, 2008; Demarque and Spitzer, 2010) and longer than what has been reported for the embryonic zebrafish spinal cord (Warp et al., 2012; Plazas et al., 2013).

### Global pharmacological modifications of the dynamic of calcium transients

To assess the ability of our pharmacological treatments to modify ENE, we measured their impact on Ca^2+^ signaling. We performed time-lapse recordings 2–10 h after the addition of the treatments to the embryo medium. Here again, the results obtained were similar in the three genetic backgrounds. Following veratridine treatment, the four parameters analyzed were increased (Fig. 1*D–O*, green symbols). In contrast, following TCNF treatments, three of the analyzed parameters were decreased, frequency, incidence, and amplitude while the duration was increased (Fig. 1*D–O*, red symbols).

These results demonstrate that bath-mediated pharmacological treatments were able to change the incidence, frequency, and amplitude of spontaneous Ca^2+^ transients in the embryonic zebrafish forebrain. When analyzing the late consequences of these transient treatments, we refer to the veratridine treatment as “increased ENE,” and to TCNF treatment as “decreased ENE.”

### Dopaminergic cells in the telencephalon at larval stages

Next, we studied the effect of the transient perturbations of ENE on the maturation of the telencephalic dopaminergic (Tel-DA) neurons distributed as two neighboring subpopulations, in the subpallium (SP), and in the olfactory bulb (OB).

To identify dopaminergic neurons in the telencephalon of 6- to 7-dpf larvae we used anatomic landmarks such as the position of brain ventricles and large fiber bundles combined to two markers of the catecholaminergic phenotype: the enzyme limiting the synthesis of catecholamines (i.e., dopamine, adrenaline, and noradrenaline), tyrosine hydroxylase (TH), and the transporter accumulating monoamines inside exocytotic vesicles, vesicular monoamine transporter (vMAT). Indeed, in zebrafish, like in mammals, adrenaline and noradrenaline-containing neuronal soma, labeled by the same markers, are not detected anterior to the midbrain-hindbrain boundary, rather, they are restricted to neuronal subpopulations located in the locus coeruleus, the medulla oblongata and the area postrema (Ma, 1994a,b 1997, 2003). Thus, all the catecholaminergic neurons located anterior to the midbrain-hindbrain boundary are likely dopaminergic neurons.

In zebrafish, TH is encoded by two paralogous genes (*th*; previously known as *th1* and *th2*). *th2* is mostly expressed in the caudal hypothalamus and is very rare in the nuclei studied here (Panula et al., 2010). We, therefore, used an anti-TH antibody that identifies Th-expressing cells (referred to as TH1^+^ cells in the remaining manuscript) but does not recognize Th2 (Yamamoto et al., 2011). vMAT is encoded by two different genes (*slc18a1* and *slc18a2*, also known as *vmat1* and *vmat2*), but only the latter is detected in the zebrafish brain (Puttonen et al., 2017), so we used an anti-GFP antibody to amplify the endogenous GFP signal from cells in the *Tg(Et.vMAT2:eGFP)* transgenic line (referred to as vMAT2^+^ cells in the remaining of the manuscript).

**Figure 2.**
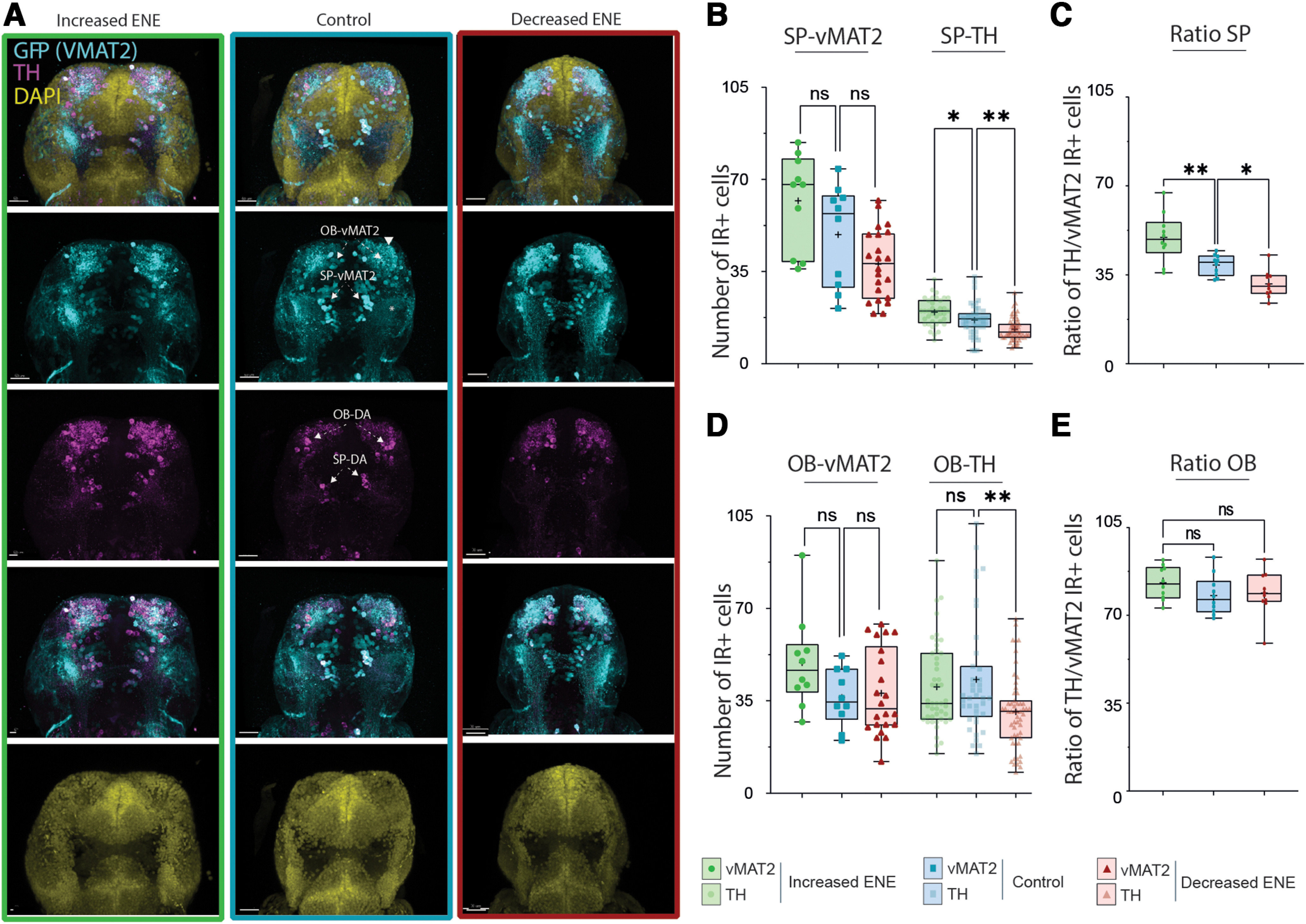
Effects of ENE on the expression of dopaminergic markers in the zebrafish larval subpallium and olfactory bulb. ***A***, Maximum projection of confocal z series of the brain of 6- to 7-dpf *Tg(Et.vMAT2:eGFP)* larvae, following ENE increase (left column, boxed in green), in control conditions (middle column, boxed in blue), and following ENE decrease (right column, boxed in red). Immunostaining to TH (magenta) and GFP (cyan) and DAPI (yellow) are shown as composite image (top) as well as for each color channels. Scale bars = 50 μm. ***B–E***, Boxplots showing the number of cells IR^+^ for dopaminergic markers in the SP (***B***) and the OB (***D***) from six independent experiments, in control conditions (blue), following ENE increase (green) and following ENE decrease (red). ***B***, Left, Number of SP-vMAT2^+^ cells, KW test, 6 < *n* < 16. Right, Number of SP-TH^+^ cells, KW test, 34 < *n* < 49. ***C***, Proportion of vMAT2^+^ cells being also TH1^+^ in the SP, ANOVA test, *n* = 24. ***D***, Left, Number of OB-vMAT2^+^ cells, KW test, 6 < *n* < 16. Right, OB-TH^+^ cells, KW test, 34 < *n* < 49. ***E***, Proportion of vMAT2^+^ cells being also TH1^+^ in the SP, ANOVA test, *n* = 24. *p < 0.05, **p < 0.01, ns = non significant.

In the SP, the cell bodies of vMAT2^+^ cells (hereafter noted SP-vMAT2^+^ cells) were distributed bilaterally along the midline, and relatively close to it (Fig. 2*A*, cyan labeling). The majority of fibers we could detect projected first ventrally and then laterally, joining the wide dopaminergic lateral longitudinal tracts.

In the OB, the cell bodies of vMAT2^+^ cells (noted OB-vMAT2^+^ cells) were distributed at the anterior end of the forebrain and were projecting anteriorly before rapidly branching in multiple directions.

In both regions, the overall disposition of the TH1^+^ cell bodies and projections were similar as for vMAT2^+^ cells. All TH1^+^ cells were also vMAT2^+^, while some vMAT2^+^ cells were TH1^−^ (Fig. 2*A*, magenta labeling). In the SP, vMAT2^+^/TH1^−^ cell bodies were mostly located at the anterior end of the cluster of vMAT2^+^/TH1^+^ cells. Such vMAT2^+^/TH^−^ cells are also detected in the SP of adult zebrafish (Yamamoto et al., 2011). The proportion of TH1^+^ cells over vMAT2^+^ cells was 38.9 ± 2.9% in the SP and 74.2 ± 3.1% in the OB (Fig. 2*C*,*E*).

### Effects of modification of ENE on the expression of dopaminergic markers in the telencephalon and the OB

To assess the effects of ENE on the expression of the dopaminergic markers in the telencephalon at larval stages, we counted the number of vMAT2^+^ and TH1^+^ cells in larvae fixed at 6–7 dpf, following the transient pharmacological treatments intended to increase or to decrease ENE.

In the SP, enhancing ENE from 48 to 72 hpf increased the number of TH1^+^ cells detected while lowering ENE decreased the number of TH1^+^ cells (Fig. 2*A*,*B*). In both experimental situations, the number of vMAT2^+^ cells did not change significantly despite a similar trend. Again, all SP-TH1^+^ cells also exhibited vMAT2 labeling. The proportion of TH1^+^ cells over vMAT2^+^ cells increased to 49.7 ± 6.8% following increase of ENE and decreased to 31.5 ± 3.9% following decrease of ENE, in both cases a significant difference from controls (Fig. 2*A*,*C*). Hence, according to our identification criteria (anatomic position combined with the expression of both TH1 and vMAT2), increasing embryonic electrical activity results in more SP-DA cells in larvae, while decreasing it reduces this population.

In the OB, increasing ENE from 48 to 72 hpf did not change the number of TH1^+^ cells detected while decreasing ENE significantly reduced the number of TH1^+^ cells (Fig. 2*A*,*D*). As in the SP, all TH1^+^ cells exhibited vMAT2 labeling, independently of the treatments. Meanwhile, the number of vMAT2^+^ cells did not change significantly in this region (Fig. 2*A*,*D*). The proportion of TH1^+^ cells over vMAT2^+^ cells did not change as well (Fig. 2*E*). Overall, our results indicate that the number of dopaminergic (TH1+) neurons in the larval telencephalon is positively affected by electrical activity during the embryonic stage. Modulating ENE has a much weaker impact on the size of the monoaminergic (vMAT2^+^) subpopulations. This suggests that ENE influences the specification of monoaminergic precursors during an embryonic critical window, driving them toward the dopaminergic fate.

### Modifications of ENE have no effect on apoptosis in the forebrain

**Figure 3.**
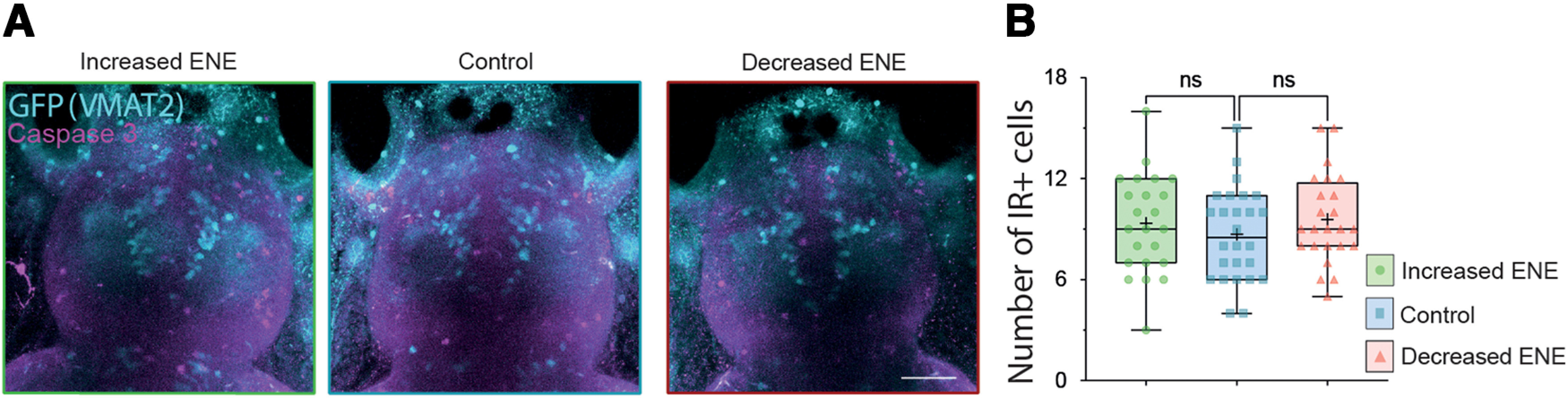
Absence of changes in programmed cell death following modifications of ENE. ***A***, Maximum projection of confocal z series of the brain of 6- to 7-dpf *Tg(Et.vMAT2:eGFP)* larvae, following ENE increase (left image, boxed in green), in control conditions (middle image, boxed in blue), and following ENE decrease (right image, boxed in red). Immunostaining to caspase-3 (magenta) and GFP (cyan) are shown as merged channels. Scale bars = 50 μm. ***B***, Boxplots showing the number of the caspase-3^+^ IR cells counted in the telencephalon following ENE increase (green), in control conditions (blue) and following ENE decrease (red). KW test, 23 < *n* < 26 telencephalon from four independent experiments. ns = non significant.

To check whether these changes in the number of dopaminergic neurons were associated with programmed cell death, we counted the number of cells immunoreactive for activated caspase-3, an apoptosis marker, in the vicinity of vMAT2 expression in the SP and OB, at 6–7 dpf. The number of caspase-3^+^ cells was stable following the increase or the decrease of ENE (Fig. 3). This result strengthens the hypothesis that the changes observed in the number of TH1^+^ cells are indeed linked to changes in neurotransmitter specification rather than changes in cell survival.

### Effects of pharmacological treatments on the locomotion of 6- to 7-dpf larvae

To analyze the consequence of the modifications of ENE on behavior, we focused our analysis on spontaneous locomotion.

During locomotion, zebrafish larvae display successive turn and swim bouts called episodes, interleaved with resting periods (Budick and O’Malley, 2000).

**Figure 4.**
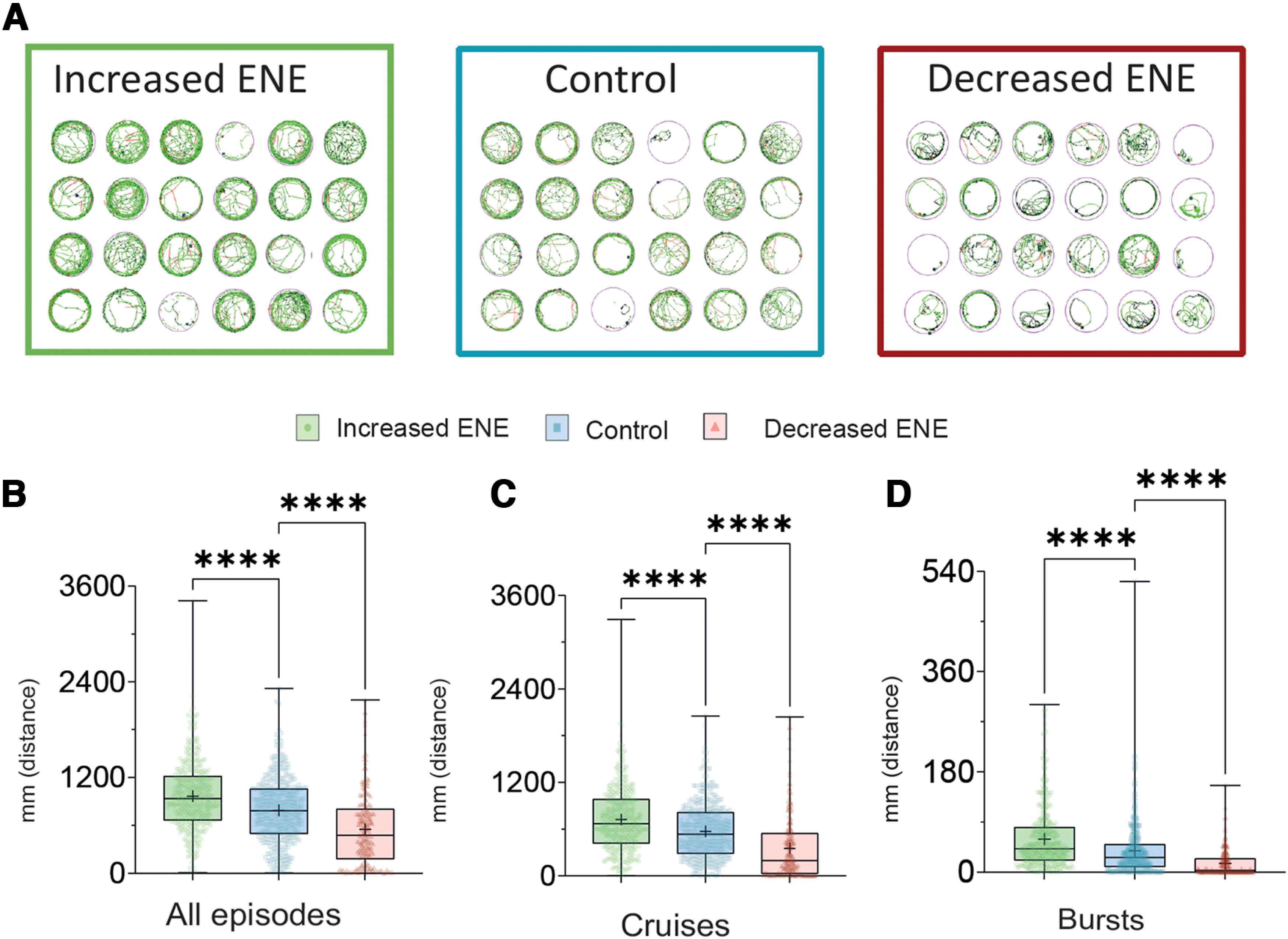
Effects of a 24-h bath applied treatments during embryonic development on spontaneous swimming of zebrafish larvae. ***A***, Representative path reconstructions for 24 individual larvae during a 10-min trial for three experimental conditions. We performed the experiments at 6–7 dpf when the properties of swimming episodes are relatively stable in control conditions. The portion of the path corresponding to cruises episodes are shown in green, and to bursts are shown in red. ***B–D***, Boxplots showing the mean distance per larva following ENE increase (green), in control conditions (blue), and following ENE decrease (red). Distance is expressed as the cumulated length of the path covered. ***B***, Distance covered when no threshold is applied for the analysis. ***C***, Distance covered during cruises. ***D***, Distance covered during bursts. For all boxplots the p of normality test for at least one condition was <0.05; therefore, KW test was used, 192 < *n* < 384. ****p < 0.001.

We compared locomotion parameters recorded at 6–7 dpf, >3 d after the end of ENE modifications (Fig. 1*A*). We recorded spontaneous locomotion of individual larvae over three consecutive 10-min sessions in each condition: control, increased ENE, and decreased ENE. A representative example of traces obtained for 24-well plates in each condition is shown in Figure 4*A*.

Increasing ENE increased the total distance covered by each larva (Fig. 4*B*, green symbols). Decreasing ENE led to a decrease of the total distance covered by each larva (Fig. 4*B*, red symbols).

Following this observation that modifications of ENE induced changes in spontaneous larval locomotion, we analyzed further these effects by discriminating two types of episodes based on their speed: slow and fast swimming episodes, dubbed cruises and bursts, respectively (see Materials and Methods; Budick and O’Malley, 2000; Kalueff et al., 2013). Increasing ENE increased the distance covered during both cruises and bursts (Fig. 4*C*,*D*, green symbols). Reciprocally, decreasing ENE, decreased the distance covered during both cruises and bursts (Fig. 4*C*,*D*, red symbols).

Of note, the distance covered during events with a speed below our low threshold did not change over the three experimental conditions (data not shown). Overall, increasing ENE increased locomotion while decreasing ENE decreased locomotion. Although the distinction between bursts and cruises may be viewed as arbitrary, the overall positive correlation between ENE and global larval activity (e.g., total distance swam) is robust.

### Washout kinetics of pharmacological treatments assessed using locomotion parameters at 6–7 dpf and calcium signaling at 2- to 3-dpf embryos

To exclude a direct contribution of the treatments applied in the embryonic period on the parameters measured in the larval period, we assessed the reversibility of the treatments by following the time course of the effects of acute pharmacological treatments in two sets of experiments.

**Figure 5.**
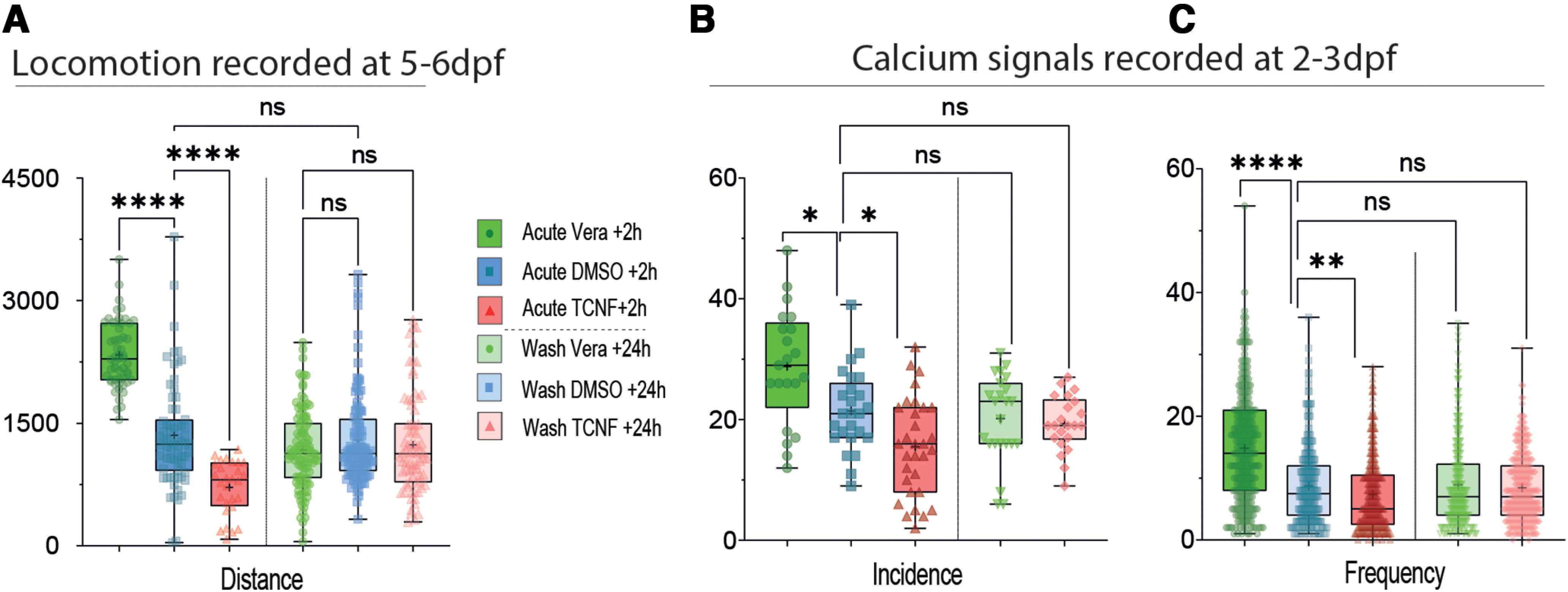
Washout kinetics of the pharmacological treatments in the larval and embryonic zebrafish brain. ***A***, Boxplots showing the overall mean distance per larva from 5-dpf larvae, following acute application of veratridine (green), in control conditions (blue), following acute application of TCNF (red) and after 24–48 h of wash for each treatment (light boxplots). For all boxplots the p of normality test for at least one condition was >0.05, therefore KW test was used, 28 < *n* < 117 fish from three independent experiments. ***B***, ***C***, Boxplots showing parameters of Ca^2+^ transients in the telencephalon of 2-dpf embryos, following acute veratridine application (green), in control conditions (blue), following acute TCNF application (red) and following 6–10 h of wash for each treatment (light green and light red boxplots). ***B***, Incidence of Ca^2+^ transients. BFW ANOVA test. ***C***, Frequency of Ca^2+^ transients, KW test. 258 < *n* < 318 values from three independent experiments. *p < 0.05, **p < 0.01, ****p < 0.001, ns = non significant.

First, on 5-dpf larvae, we performed 10-min recording sessions and analyzed the overall distance covered before the application of the treatments, then +2 h after the application of the treatments, and lastly 24 h after washing out the drugs. Acute treatments of swimming larvae had the expected effects: veratridine application induced an increase in the distance covered, while TCNF application led to a decrease in the distance covered (Fig. 5*A*, left boxplots). By the next day, all the effects of the treatments on burst episodes had faded away demonstrating the washout properties of the pharmacological agents used (Fig. 5*A*, right boxplots).

Second, we recorded the washout kinetics of pharmacological treatments on Ca^2+^ signals recorded in 2- to 3-dpf embryos. We applied the drugs at 48 hpf, as before. We recorded 20-min sessions between 2 and 4 h after the beginning of the applications, then washed the treatments, waited for 6–10 h, and perform new 20-min sessions (Fig. 5*E*,*F*). As described in Figure 1, after 2 h of treatment, the incidence and frequency of Ca^2+^ transients were increased by veratridine and reduced by TCNF (Fig. 5*E*,*F*, left boxplots). For both treatments, the values measured after the washing time (or washout) were back to control values (Fig. 5*E*,*F*, right boxplots).

These results indicate that washout of veratridine or TCNF results in rapid loss of direct effect of the drugs, in a few hours at most. Thus, the timing of our analysis of larval locomotion, 3 d after ENE manipulation, is far beyond the time required for the disappearance of the acute effect of the drugs. Hence, our results support the existence of a long-term effect related to changes in neural excitability during the embryonic period in the zebrafish.

## Discussion

### Summary

Immature forms of neural excitability, ENE, include spontaneous Ca^2+^ transients, activation of nonsynaptic receptors to neurotransmitters and early synaptic activities (Ribera and Spitzer, 1998). Our results show that the developing zebrafish is a suitable model for manipulating ENE through the use of pharmacological treatments and for studying the consequences of these manipulations on the plasticity of neuromodulator systems and their behavior. Indeed, we report that ENE exerts a positive regulatory effect on the specification of the dopaminergic phenotype by increasing the number of dopaminergic neurons in the subpallium as well as high-speed swimming episodes.

### Bath application of pharmacological treatments and ENE

For the present study, zebrafish embryos were exposed to pharmacological compounds by bath application. The zebrafish is highly suitable for such questions because of its external development in an egg. In addition, our washout experiments agree with data in the literature indicating that the blood-brain barrier starts to form around 72 hpf but is not fully mature until 10 dpf (Fleming et al., 2013), allowing the diffusion of drugs to neural tissues during treatments and their subsequent removal on washing at embryonic stages. This methodological approach induces both global and transient effects. It helps assess the net effects of the treatments on different subpopulations of cells, in different regions of the animal. Further studies using more localized modifications of ENE could help decipher the contribution of specific brain regions.

We focused on Ca^2+^ transients, one of the main forms of immature excitability, to assess the effectiveness of the treatments. However, other intercellular communication processes are also likely perturbed by the treatments, such as the paracrine activation of GABA and glutamate receptors by endogenous transmitters, and initial synaptic activities. Further studies are required to decipher the specific contribution of each of these mechanisms to the cellular and behavioral effects we reported here.

### Calcium transients in the zebrafish subpallium

One reason to focus on Ca^2+^ transients is that they are conveniently measured by Ca^2+^ imaging. In addition, they have been previously involved in neurotransmitter phenotype specification in other models of neuronal plasticity. For example, in the developing *Xenopus* spinal cord, these Ca^2+^ transients induce the release of brain-derived neurotrophic factor (BDNF), which result in noncell-autonomous phenotype switching (Guemez-Gamboa et al., 2014). We chose to drive the expression of GCaMP ubiquitously at first to ensure we would not miss relevant cell populations at the selected period of recordings. Further studies using a pan-neuronal promoter revealed very similar patterns, leading us to conclude that activity recorded in neurons are likely accounting for the majority of the calcium pattern observed. Surprisingly, the number of observed reactive cells was actually higher with the neuronal promoter, possibly because of a higher expression of the transgene in this line. The next step would thus be to use promoters of different cellular subpopulations, in particular markers of the DAergic lineage to identify the differentiation status of the cells where the Ca^2+^ transients are detected. It would also allow us to see whether there are different dynamics of Ca^2+^ transients in different subpopulations of DA neuron precursors.

Ca^2+^ transients were already reported in the spinal cord of zebrafish embryos around 24 hpf (Warp et al., 2012; Plazas et al., 2013). Here, recordings were performed in the subpallium and olfactory bulb of embryonic zebrafish at around 48 hpf, a time at which the transition toward synaptic network activity has already occurred in the spinal cord (Warp et al., 2012). However, the long duration of the transients we measure is clearly consistent with ENE rather than classical synaptic activity, and consistent with the fact that telencephalon is the site where synaptic maturation occurs last (Goode et al., 2021). These observations are in agreement with the existence of a postero-anterior gradient of neuronal maturation, similar to what was described in *Xenopus* (Papalopulu and Kintner, 1996).

The duration of Ca^2+^ transients we measured in the subpallium (7.8 ± 2.5 s) were similar to what has been reported in *Xenopus* (7.5 ± 1.0 s; Gu et al., 1994) and longer than what has been described in the zebrafish spinal cord (3.3 ± 1.6 s; Plazas et al., 2013). The duration of these individual transients was increased by both the veratridine and the TCNF treatments reported here, while these treatments led to diametrically opposite effects on DA specification and high-speed locomotion. This suggests that the duration of the transients is not necessarily the pertinent signal to trigger the effect on neuronal differentiation. Frequency, and possibly amplitude, of transients appear to be more relevant candidate specification triggers.

### Potential mechanisms linking changes in ENE and plasticity of the neurotransmitter phenotype

Several mechanisms underlying the contribution of activity-dependent processes in the choice of neurotransmitter phenotype have been proposed (Li et al., 2020). For neurons in the dorsal embryonic spinal cord of *Xenopus* tropicalis, a direct link between endogenous Ca^2+^ transients and an intrinsic genetic pathway has been shown to influence neurotransmitter choice. Ca^2+^ signals increase the phosphorylation of the transcription factor c-Jun. In turn, phosphorylated c-Jun drives the expression of another transcription factor, tlx3, which favors the GABAergic fate over the glutamatergic fate. This process contributes to homeostatic plasticity of the neurotransmitter phenotype specification (Marek et al., 2010).

A list of transcription factors involved in the specification of the zebrafish dopaminergic phenotype expressed in dopaminergic neurons or in their vicinity at 96 hpf is available (Filippi et al., 2012). Some may have an expression that is sensitive to the level of activity, providing critical decision points in genetic networks for neurotransmitter specification. To test this hypothesis, the sensitivity to the activity of the expression of several of these candidates will be tested in the future.

Indirect mechanisms may also be involved. Ca^2+^ transients have been shown to induce the release of BDNF, which in turn triggers the activation of the TrkB/MAPK signaling cascade that modulates the expression of transcription factors involved in neurotransmitter fate selection (Guemez-Gamboa et al., 2014).

In ecotoxicology, following exposure to modulators of activity, both direct and indirect mechanisms are likely activated and further analysis is required to disentangle their respective contributions to the phenotype plasticity reported here.

### A critical period for the effect of ENE perturbations

We observed an effect of the pharmacological treatments performed during the embryonic period on spontaneous locomotion several days after the end of the treatments. In contrast, no effects were observed 24 h after treatments performed at 5 dpf in the zebrafish larvae. These long-lasting effects of treatments specifically during the embryonic period are in line with the existence of a “critical period” during development, which is more sensitive to homeostatic perturbations and promotes significant phenotypic plasticity in immature neurons. Since dopaminergic systems have multiple functional roles and are particularly prone to plasticity events (Collo et al., 2014; Macedo-Lima and Remage-Healey, 2021), this suggests that these systems might be a key factor for the adaptability of animals to environmental changes. Whether it is a cause, a consequence, or a simple correlation of their conservation throughout animal evolution is still an open question.

### Identification of a biomarker for DA reserve Pool neurons

SP-DA neurons are not present in *Xenopus* and to the best of our knowledge, the plasticity of their dopaminergic phenotype has not been studied so far. In the SP, the effect of ENE perturbations on the DA phenotype analyzed by the number of TH1^+^ cells was in agreement with the homeostatic rule described in *Xenopus* (Spitzer, 2012). Indeed, the DA phenotype was enhanced by increased excitability, and decreased by decreased excitability, as expected from an overall inhibitory neurotransmitter. In the OB, we observed a similar decrease of the DA phenotype following decreased excitability but no increase following increased activity. This is in contradiction to what has been reported in *Xenopus* (Velázquez-Ulloa et al., 2011). This unexpected result could be because of the number of TH1^+^ cells already set at a maximum in basal conditions in the zebrafish OB as suggested by the high ratio of vMAT2^+^/TH^+^ observed in this region compare to SP in control conditions.

The relative stability of the number of vMAT2^+^ cells, together with no change in caspase 3-labeled cells on treatments, suggest that modulation of excitability did not affect cell death or proliferation, but rather influenced the commitment of precursors toward a dopaminergic fate. This plasticity of the DA phenotype is likely to occur within the pool of vMAT2^+^ cells since the number of vMAT2^+^/TH^+^ was increased with increased excitability. Therefore, the vMAT2^+^/TH^−^ cells could be a reserve pool of cells primed to become dopamine neurons when plasticity-triggering events occur. The expression of the vesicular transporter in a reserve pool of cells might have a functional advantage in terms of response to plasticity-triggering events, limiting the number of factors to be changed for reaching a fully functional dopamine phenotype.

### Behavioral consequences of ENE

Perturbations of ENE had also behavioral consequences in zebrafish larvae, modifying spontaneous locomotion. The more ENE, the higher the distance covered fitting with a contribution of dopamine. This distance was modulated for both slow and fast swimming modes (cruises and bursts), although these two behaviors are believed to involve distinct networks, suggesting that ENE should modulate a common regulatory circuit. Determining whether direct activation of dopaminergic neurons in the subpallium could stimulate cruises or bursts using optogenetic tools is an open perspective for the future. It would be interesting to test whether other behaviors such as prepulse inhibition are also affected following alterations of ENE.

### Potential mechanisms linking changes in ENE and changes in locomotion

Links between movement control and the neuromodulator effect of dopamine are widely described. In zebrafish, it has been involved in the initiation of movement (Thirumalai and Cline, 2008), via direct projection from dopaminergic cells in the diencephalon (Lambert et al., 2012). Dopaminergic cells in the hypothalamus also modulate locomotion (McPherson et al., 2016). The global perturbations we used likely induced plasticity mechanisms beyond the dopaminergic cells we analyzed in this study. Changes within the spinal networks itself, and nondopaminergic modulatory networks probably occurred as well, preventing us to conclude about a causal link between the number of telencephalic DA neurons and the level of spontaneous locomotion, the two phenotypes we report here.

### Perspectives: transcriptomics and model to test the impact of environmental factors on ENE

The developing zebrafish provide an attracting model in which to perform transcriptomic analysis to identify genes differentially expressed in vMAT2 cells following modifications of ENE. The results would help decipher the cellular and molecular mechanisms underlying the modulatory role of ENE during brain differentiation.

The data presented here point to an implication of ENE in the regulation of dopaminergic differentiation in the developing brain, suggesting that ENE could act as an intermediate between environmental factors and the molecular changes leading to the alteration of dopamine-related behaviors. The zebrafish model is also adapted to test how environmental factors such as malnutrition, stress, and drug exposure could modulate ENE and potentially influence the maturation of the dopaminergic systems.

## Synthesis

Reviewing Editor: Nathalie Ginovart, Universite de Geneve Decisions are customarily a result of the Reviewing Editor and the peer reviewers coming together and discussing their recommendations until a consensus is reached. When revisions are invited, a fact-based synthesis statement explaining their decision and outlining what is needed to prepare a revision will be listed below. The following reviewer(s) agreed to reveal their identity: Matt Parker.

The original two reviewers still diametrically differed in their overall assessment of the resubmitted manuscript. Although both agreed that this version was improved, reviewer 1 still identified several shortcomings and methodological concerns, which were either not satisfactorily addressed or were ignored following the first round of review. The manuscript was sent to a third reviewer so as to get an additional opinion before making a final decision. The third reviewer found the manuscript interesting but shared the same concerns with reviewer 1 regarding methodological issues and interpretation of the data and confirmed that these must be addressed. The resubmitted manuscript is therefore not acceptable as is and we all agreed that it is essential that the authors respond properly to the critical comments of reviewers 1 and 3 before the article can be accepted for publication in eNeuro.

Please find the specific comments and queries raised by the reviewers below:

Reviewer 1:

The authors have made changes to the manuscript, which is somewhat improved. Some questions have been addressed well, such as the washout studies. However, some of the concerns have not been addressed satisfactorily.

Review synthesis:

1. In response to “In contrast to what is stated by the authors, TH-IR is not statistically increased in the olfactory bulb” the authors now state that “We used an R protocol to remove outliers over and below the 5th and 95th percentiles [it] allowed to reach statistical significance.” This is not a rigorous or acceptable way to handle the data and has likely led to spurious statistical significance.

2. In response to “the authors should present the proportions of VMAT2+/TH+ and VMAT2+/TH- cells” the authors have not made this change, stating that it is not technically possible to do.

Reviewer comments

1. In response to “it is presumed that the ’spikes‘ represent intrinsic or non-synaptic cellular depolarizations, although it is really not clear what causes these events” the authors now state that “calcium spikes have been long described in the xenopus central nervous system before synapses are detectable”. This is probably correct, but the experiments here are done in zebrafish. Basing the interpretation of the findings on studies done in a different species from a different class of the vertebrata subphylum does not seem a sensible approach. It seems rather improbable that there are no synapses at the developmental point examined here, in view of the appearance of coordinated swimming behavior in zebrafish at around this age (for example doi:10.3791/54431). In the absence of compelling supportive data, the authors should not conclude that the origin of the transients is intrinsic or paracrine; synaptic activity has not been excluded.

2. In response to: “the heat shock promoter is ubiquitous, so even within the neural tube is likely to be expressed in multiple cell types other than neurons” the authors now state that “The analysis of calcium transients dynamics was performed blind, independently of the glial or neuronal nature of the recorded cells” and include a section in results stating that “we used ubiquitous reporters to detect changes in fluorescence in telencephalic cells of different nature including putative differentiating dopaminergic neurons, non-dopaminergic neurons and glial cells”. Since it is now clear that the recordings include cells other than neurons, it is inaccurate to describe the resulting data as showing “embryonic neuronal excitability” and this should be revised.

3. Was there a good reason for not using a neuronal driver to express GCaMP, several of which are available that express at this time point? This would have avoided the ambiguity about cell types of origin.

4. In response to: “The arbitrary division of movements into cruises and bursts is not well-justified” and the reviewer’s subsequent explanation, the authors state that “this is a serious concern that we share with the reviewer”. The complete dataset is now shown without arbitrary quantitative constraints - and notably, many of the apparent differences seen after arbitrary categorical division of the data disappear in the complete dataset. Inexplicably, however, the authors still show the data divided arbitrarily into categories and analyzed separately. There seems to be little neurobiological basis for categorically assigning movements based on arbitrary quantitative thresholds in this way, and it is recommended this is removed. Please also see #6 below, as, depending on how the analyses were done, there is potentially a very significant problem with using swimming speed to make categorical assignments based on the distribution within each experimental group (so that a different range of swim speeds is included in the categorized datasets from different groups), and then comparing swim speed between groups within each arbitrary category.

5. Questions are raised by the additional methodological details now provided. The definition of movement events was anything above (mean speed)/2, “This low threshold was set in order to eliminate activity that did not contribute to the behavioural phenotype, such as small changes in orientation.” This accounts for the abrupt cutoff and unusual shape of the lower end of the data distributions in figure 4E, but it is unclear how this threshold was chosen or validated, particularly when many of the movements still seem to have duration or distance close to 0. It is conventional to use thresholds to distinguish true movements from pixel noise in the video stream, but it is not clear this was achieved here, and it is also unclear what movement data were eliminated by this process. It is also not explained why the authors feel that orientation changes are not part of the motor phenotype, since these are a predominant movement type in constrained behavioral arenas.

6. The revised figure 4 raises other questions. There are no units in the graphs or figure legends for figure 4. Is each point a different zebrafish or a different movement? The number of events would be expected to be reported per zebrafish, but the duration, distance and speed of events seem to have the same number of points suggesting they are mean values for each zebrafish. Were the thresholds for assigning movements to burst or cruise calculated and applied per group, per fish or for the population overall? This needs to be clarified, although it is suggested that the arbitrary movement categorizations are removed.

7. In response to “no evidence is provided to link these differences in motor function to the changes in the dopaminergic system shown in the preceding figures” the authors now state that “We were not able so far to perform the experiments linking both observations and to the best of our knowledge, there is no claim in the manuscript that we did.” A series of unrelated observations without any mechanistic link is descriptive and does not provide a conceptual advance.

The manuscript still makes claims that are not substantiated by the data in the paper.

1. “hyperlocomotion, a phenotype associated with ADHD and schizophrenia in this model” requires so much qualification that it is incorrect as stated. There is no evidence that anything in this dataset relates directly to any human disease.

2. “conditions close to physiological exposure and ecotoxicology” is also not supported by the data - the reviewer would be interested to learn where zebrafish are physiologically or ecologically exposed to a cocktail of channel blockers up to 10μM in their water; this is just implausible.

3. “open important perspectives for this model to decipher the chain of events leading from environmental factors to the pathogenesis of brain disorders such as schizophrenia and ADHD” is an extreme stretch on multiple levels and should be removed.

Other points

1. Please use the correct gene terminology. Zebrafish genes are conventionally notated in lower-case italics, thus <i>vmat2</i> not vMAT2 as written here. The correct terminology for the two TH paralogs is <i>th</i> and <i>th2</i> (gene) and Th and Th2 (protein). Please read the section about zebrafish nomenclature on ZFIN.org

2. “Balneation” still appears in some figure legends, suggest replacing with “bath application” to match the rest of the manuscript.

Reviewer 2 :

The manuscript entitled “Plasticity of the dopaminergic phenotype and of locomotion in larval zebrafish induced by changes in brain excitability during the embryonic period” investigates the role of embryonic neuronal excitability on the specification of the dopaminergic phenotype in subpallium and olfactory bulb neurons and on the locomotor behavior of developing zebrafish larvae. The authors measured the spontaneous neural activity by expressing GCaMP6f and time-lapse imaging these structures in zebrafish embryos. The study shows that by incubating embryos with ion channel agonist and antagonists during a critical embryonic period, neural activity is enhanced or inhibited which results in changes in the number of dopaminergic neurons and in the swimming behavior of zebrafish larvae.

This is an interesting study that contributes to advance the field of activity-dependent neural development by demonstrating a role for embryonic neuronal activity on the specification of neurotransmitter phenotype in zebrafish brain and the consequences to relevant behavior for this species. This, in turn may contribute to the understanding of the mechanisms of neurodevelopmental disorders in humans.

The authors satisfactorily addressed all my concerns raised in the original submission.

Minor points that need to be revised:

1) In line 473, replace Figure 2C with Figure 2B.

2) In line 487, replace Figure 2E with Figure 2C.

3) In line 616, after episodes (end of sentence) add reference to Extended Figure 4-1.

4) On the argument of the developmental delay or acceleration in locomotor function when spontaneous embryonic activity is manipulated, I think the authors need to add to that paragraph in pages 26 and 27, whether larva’s gross development was comparable among control and treated groups at 6-7 dpf. If that was the case, then I don’t see the point of discussing whether there is a delay or acceleration in locomotor function development. Because the point is that those manipulations did not affect overall larval development but particularly affected the locomotor behavior, whether it was through interfering with the normal developmental rate of this function or not is not addressed with this set of experiments and is also not relevant to focus of this study.

In my opinion, Extended Figure 4-1 is either not needed or could be presented to illustrate the normal development of locomotor behavior, but not as an explanation that is not a shift in developmental rate.

5) The manuscript would benefit from additional editing to correct remaining grammar issues in some sentences.

Reviewer 3 :

This is an interesting article that could potentially advance the field. However, there are several methodological weaknesses that need to be addressed.

1) there are several places in the paper where the authors over-interpret/claim about translational relevance to human conditions, without any real data to back this up (eg suggesting this is a model for ADHD/Schizophrenia). As a basic science paper this is fine - please don’t oversell (with nothing to support)

2) There are several concerns about the methodology raised by the original reviewer that remain unanswered- these need to be addressed and not ignored. eg the extrapolation from xenopus to zebrafish with respect to the calcium spikes needs to be reconsidered.

3) behavioural data with respect to movement is not clearly about the calculations. How was this validated?

## Author Response

We first would like to thank the editor and the reviewers for their feedback on our manuscript eN-NWR-0320-21. We also would like to thank eNeuro and its editorial associate for granting us an extended period to address the comments to take into account the move of the Paris-Saclay Neuroscience Institute to a new building in Saclay during the revision period.

Please find below, in blue, our comments on the issues raised in the reviews.

Synthesis of Reviews:Synthesis

Statement for Author (Required):

The reviewers diametrically differed in their overall evaluation of the present manuscript.

Whereas one reviewer expressed minor concerns, the other reviewer raised serious concerns pertaining to methodological issues, including calcium imaging and the use of heat shock, as well as logical flaws in the interpretation of the results that may significantly hinder the conclusion on the effect of embryonic calcium-dependent activity on neurotransmitter specification. Importantly, both reviewers expressed concerns about the lack of delineation of the mechanism linking changes in neuronal excitability, TH expression and motor behavior.

Other issues were raised relating to inconsistencies between the results description and the figures. Upon a thorough discussion with the two reviewers, we agreed that the manuscript cannot be accepted in its present form. There was also a consensus that electrophysiological experiments may not be mandatory provided the authors moderate their words on what they are measuring with calcium imaging. However, it is important to fully address the critical comments of reviewer 1 regarding methodological issues and interpretation of the data. I enclose below the individual comments of the reviewers to be addressed in a resubmission. In addition to their comments, please also refer to my comments below: 1 - There are major inconsistencies in the MS, notably in the results section where the description of Fig. 2 in the text does not match the data in the figure - in contrast to what is stated by the authors, TH-IR is not statistically increased in the olfactory bulb when increasing excitability, and both TH-IR in olfactory bulb and VMAT2 in telencephalon do statistically diminish when decreasing excitability with TCNF.

We used an R protocol to remove outliers over and below the 5th and 95th percentiles from the quantification of IR, as shown in S1 it doesn’t change the overall boxplots shape but allowed to reach statistical significance.

Also, the total, duration of swim bouts (4.6 {plus minus} 2.1 %, line 384) does not match the data shown in the figure.

The results in the sentence are expressed as % increase or decrease relative to control values. 2 - To support their claim that the DA phenotype occurs within the pool of VMAT2+ cells, the authors should present the proportions of VMAT2+/TH+ and VMAT2+/TH- cells at control conditions and following changes in neuronal excitability.

Unfortunately the experiments were recorded sequentially for most for them. The number of experiments with both stainings is not important enough to conclude on the ratios. We moved the hypothesis of a reserve pool identified by vmat2 staining to the discussion section, with a more speculative tone.

Reviewer 1:

This study describes the phenotypic effects of immersing larval zebrafish at 48 - 72 hpf in chemicals that (i) prevent voltage-gated sodium channel inactivation (10uM veratridine), or 2 (ii) block voltage-gated sodium (TTX 2uM) and calcium (conotoxin 0.08uM, nifedipine 0.4uM, flunarizine 2uM) channels. Four phenotypic outputs are reported: (i) calcium imaging at 48 - 72hpf; (ii) number of VMAT+ neurons that also express TH at 6 - 7dpf; (iii) Caspase-3 expressing cells at 6 - 7dpf; (iv) motor function at 4 - 7 dpf. The principal conclusions are that exposure to the chemical agents at 48 - 72hpf causes immediate alterations in cytosolic calcium and later changes in TH expression and motor activity.

The concept that the physiological activity of neurons during development influences the course of development and thereby alters outcomes after development is not new, although the question has not been explored in this model in exactly this way previously, and the work may be of potential interest to developmental neurobiologists.

There are several major weaknesses in the work as presented:

1. In figure 1, it is presumed that the ‘spikes’ represent intrinsic or non-synaptic cellular depolarizations, although it is really not clear what causes these events. ‘Spikes’ is also a misleading term, as the events last 3 orders of magnitude longer than action potentials (it is generally accepted from paired imaging/patch clamp studies that only long trains of action potentials or prolonged depolarization events cause sufficient change in intracellular calcium to be detectable by genetically encoded calcium sensors).

The presumption that these events are not driven by synaptic transmission is not necessarily correct. Additional studies using chemicals that block synaptic transmission and electrophysiological recordings would be necessary to substantiate these claims. Calcium spikes have been long described in the xenopus central nervous system before synapses are detectable, events shorter than 3 sec are detectable and demonstration of the ability of Gcamp6f to detect single action potentials are available. In the hope to avoid misunderstandings from a non specialist audience and to increase the clarity of the manuscript, we decided to replace the term “spike” by “transient”.

2. The use of heat shock (which is very substantial here, 38C for 90 minutes) to activate the transgene is a major weakness, since this itself causes multiple gene expression changes so the system is non-physiological.

As listed by the reviewer in his introductory paragraph, the are four lines of main results in the manuscript. Only the one related to the effects of the pharmacological treatments on embryonic neuronal excitability were performed using calcium recordings following heat-shock procideure.

The others were obtained following protocols not including heatshocks.

In order to double check that the effect of the treatments on calcium transients dynamics were not related to heatshock, we repeated the experiments usig a second transgenic line to drive the expression of the calcium sensitive probe.

To avoid timing issue related to transgene expression we selected the (gal4:TBP) line, an ubiquitous expression of the UAS reporter

3. It is not clear how neurons were identified for recording since the heat shock promoter is ubiquitous, so even within the neural tube is likely to be expressed in multiple cell types other than neurons.

The analysis of calcium transients dynamics was performed blind, independently of the glial or neuronal nature of the recorded cells. The assumption is that among the cells displaying transients few are likely differentiating dopaminergic cells.

We also do not exclude a contribution of calcium transients from other cell populations via a non cell autonomous effect (as has been shown in xenopus). 3 Identifying the nature of the cells producing the calcium signals is an open and interesting question, which may be the focus of future research in the team but is outside the scope of the current mansuscript.

4. The distinction between intrinsic cellular activity and synaptically-driven activity may be artificial anyway, as dopaminergic neurons have both, even in adult zebrafish. As natural pacemaker cells, dopaminergic neurons do not require synaptic inputs for activity even after early development is completed.

5. In figure 2, the data indicate that treatment with chemicals that enhance activity (as evaluated by calcium imaging) appears to also increase the number of VMAT cells that are also classified as TH-expressing. There are several problems with the interpretation. a. First, it is not clear that the mechanism involves a direct contribution of electrical activity to gene expression. The cells that were imaged in Figure 1 are not necessarily developing dopaminergic neurons, as the GCaMP transgene is expressed under a ubiquitous heat shock promoter, so it is unclear that the interventions actually modulated electrophysiological activity in dopaminergic neurons or that this is the cause of the gene expression changes.

As designed, the experiments do not allow to conclude whether the action of calcium transients is cell autonomous or not. If it is similar to what has been reported in xenopus it is likely non-cell autonomous. And it is indeed expected to act through modification of activity-dependent expression of transcription factors involved in fate specification acting as switch from one phenotypical outcome to another. b. Since the nervous system is interconnected and gene expression can be modulated by a range of signals, it is likely that indirect mechanisms either mediate or contribute to observations.

We fully agree with the reviewer’s comment and we believe the manuscript is not in disagreement with this statement. To strengthen this point further we added a sentence specifically related to this hypothesis in the discussion. c. Gene expression is rarely ‘all or none’ and most types of toxicant cause TH downregulation in dopaminergic neurons in a wide variety of experimental systems. The changes in TH expressing cells may simply be a change in the number of cells with expression above a threshold, in the context of non-specific downregulation of TH. In that case it would be difficult to explain the higher number of TH+ cells following veratridine treatments. The case of plasticity is possible to make in part because treatments with opposite effects on excitability have opposite effects on differentiation of the TelDA neurons and on fast swimming episodes.

6. The possibility of the intervention altering cell death has not been excluded in Figure 3, since the variation in the caspase method is substantially larger than the mean, so that extremely large changes in cell death would be necessary to see any difference in this assay, yet the differences in TH count between treatment groups are rather subtle.

We repeated the experiments to increase the consistency of the experiments. We also performed more careful analysis, focusing our counting of caspase positive cells in the region of dopaminergic markers expression (rather than in the whole region). These analysis, decreased the standard deviation of the results and the differences in numbers observed could not account for the change in the changes observed in the number of TH + cells. 4

7.The development of motor function shown in Figure 4 has been reported in multiple prior publications from different groups. Although these observational data form an essential control basis for the experiments shown in Figure 5, it is not clear how they differ from prior work.

We moved the carresponding data to a supplemental figures related to the former figure 5 (now figure 4 in the revised manuscript).

8. The arbitrary division of movements into cruises and bursts is not well-justified. These categorical assignments are apparently based on average swim speed per bout, which is influenced by the duration of the bout and the swimming velocity, so it does not seem to make sense to then analyze swim speed, distance, and duration separately in each category. A better justification for separating these events might include a bimodal distribution of relevant parameters across all events, suggesting a physiological basis for the categorical assignment.

However, this has not been found previously and the arbitrary division of cruise and burst, particularly in such a non-physiological paradigm as swimming alone in a constrained well, does not seem appropriate.

This is a serious concern that we share with the reviewer.

9. In addition, the division of quantitative data into arbitrary categories has likely artificially increased statistical power by constraining the variability that would be inherent in analyzing all movements together. This is not rigorous, especially as the effect sizes seem small and there is no justifiable physiological basis for dividing the movements into categories based on arbitrary numerical boundaries.

To address this issue, we performed the analysis on data without taking into account the threshold between cruises and bursts. We included the results in the related figure.

10. Even if it is accepted that the motor activity experiments show true differences between treatment groups, no evidence is provided to link these differences in motor function to the changes in the dopaminergic system shown in the preceding figures. Indeed, it is unclear that the dopaminergic groups counted in the telencephalon and olfactory bulb in Figure 2 play any role in motor function (prior work implicates the ventral diencephalic groups in movement). And, of course, systemic exposure to extremely high concentrations of channel blockers is likely to have induced many changes other than those observed in TH expression.

Our main conclusions are that transient perturbations of excitability restrained to the embryonic period of zebrafish development have consequences on the larvae, at the level of the dopaminergic neurons in a subset of neurons in which a plasticity of the phenotype had not been described before and at the level of the behavior. We were not able so far to perform the experiments linking both observations and to the best of our knowledge, there is no claim in the manuscript that we did.

11. It is noteworthy that the ‘long term’ changes shown in Figure 5 are similar but less prominent than the acute changes shown in Figure 6. Although one interpretation is that the chemicals caused permanent developmental changes, another is that the chemicals added early in development have not been completely eliminated at the later time points the assays are completed. This seems an important question since the biological toxins are used at very high concentrations. The authors partially address this with washout experiments at 6 - 7dpf, but the metabolism of these toxins at earlier time points may be different. To address this issue we performed a set of experiments to test the washout dynamics in the embryonic brain. We first tested the ability of brief (2 hours) application of the treatments to induce change in excitability. We then allowed for at least 4hours wash and recorded embryos 5 for the same batch and were able to report a washout following this protocol suggesting that the results are indeed corresponding to “long-term” modifications induced by the transient 24h embryonic treatments.

12. Overall, the study is descriptive and there is no delineation of mechanism. Understanding the mechanisms linking the different observations, if any, would make a much more compelling manuscript.

We have included a section to discuss the potential mechanisms involved.

13. An essential question of scientific rigor - were the data analyzed by an observer blinded to treatment group, or by an automated method that eliminates observer bias? Yes, experiments were performed blind. TH counts were performed on images files coded in numbers and the key were only communicated for the compilation of the results in the statistic software. Calcium and locomotion experiments were analyzed using semi-automated methods unbiased by the conditions.

14. Data points should be shown on graphs, as shown in Figure 4. This is not the case in figures 1, 2, and 3 where the data distribution and samples sizes are obscured.

All datas were re-analyzed in order notably to show all data points and sample sizes are now cited.

Minor points

‘Balneation’ is a Victorian word defined as “the act of bathing or the administration of public baths”. I understand that the chemicals were added to the water housing the zebrafish (bath application), but I strongly recommend replacing with standard descriptive/scientific English to avoid confusion.

We replaced balneation by “bath applied”

The zebrafish BBB starts to form at around 72hpf and so there are issues with penetration of chemicals from the bath at time points before 10dpf (discussion).

The “washout” experiments we performed address part of this comment showing that the brain tissue s indeed accessible to bath-applied treatments at that time and allow correct washout with time.

It seems an extreme stretch to suggest that exposing zebrafish embryos to high concentrations of channel blockers will elucidate the developmental molecular basis for autism and schizophrenia. It is recommended that a more measured approach is taken to the discussion, to include study limitations, explain how the findings advance the field and contextualize with prior literature.

The corresponding section in the discussion has been revised according to the reviewer’s comment. Notably we rephrase what we believe are the potential opened by the resulst in terms of studying fundamental biological mechanisms potentially at play in the pathogeneiss of neuropathies.

Reviewer 2 :

The manuscript entitled “Plasticity of the dopaminergic phenotype and of locomotion in larval zebrafish induced by changes in brain excitability during the embryonic period” investigates the role of embryonic neuronal excitability on the specification of the dopaminergic phenotype in telencephalon and olfactory bulb neurons and on the locomotor behavior of developing zebrafish larvae. The authors measured the spontaneous neural activity by 6 expressing GCaMP6f and time-lapse imaging the telencephalon and olfactory bulb of zebrafish embryos. The study shows that by incubating embryos with ion channel agonist and antagonists during a critical period, neural activity is enhanced or inhibited which results in changes in the number of dopaminergic neurons and in the swimming behavior of zebrafish larvae.

This is an interesting study that contributes to advance the field of activity-dependent neural development by demonstrating a role for embryonic neuronal activity on the specification of neurotransmitter phenotype in zebrafish brain and the consequences to relevant behavior for this species. This, in turn may contribute to the understanding of the mechanisms of neurodevelopmental disorders in humans.

Overall, the manuscript is clearly written and data are well presented and analyzed appropriately.

There are some minor issues that the authors need to address:

1) In the Introduction, page 4, line 55, the authors need to correct the species that the referred study used. Dulcis et al., 2013 was not done in mice but in rats.

The mistake has been corrected

2) The authors need to avoid redundancy by not repeating the data numbers in the text that are already represented in figures. Instead, remove all numbers from the text and add the information on statistical tests used with p and beta values in the respective figure legends.

The result section has been revised according to the reviewer’s comment.

3) In the Discussion, more references need to be included. There are several instances that the authors cite the status of the current knowledge without referring to any study. For instance, end of line 467, end of sentence in line 469, end of sentence in line 485, end of sentence in line 524, end of sentence in line 529, first half of sentence in line 541, to mention some.

Several references were included along the discussion to address the reviewer’s comment, trying to tie each state of the art information to the corresponding publication.

4) Also, the authors should elaborate on the mechanisms that lead to the type of behavioral phenotype zebrafish larvae develop when manipulating embryonic neural activity and how this can or cannot be explained by the change in the dopaminergic phenotype they observed. A paragraph dedicated to the potential mechanisms has been added to the discussion

5) In Figure 1B, add the concentration for veratridine.

The concentration for veratridine has been added to the corresponding panel
